# Transcatheter Tricuspid Valve Replacement in the Presence of Pacemaker Lead–Related Tricuspid Regurgitation and Complex Anatomy

**DOI:** 10.1016/j.jaccas.2025.105427

**Published:** 2025-10-22

**Authors:** Alexandru Patrascu, Mohammed Alkasab, Thomas Attumalil, Bryan Traynor, Kendra Derry, Sami Alnasser, Andrew Ha, Neil P. Fam

**Affiliations:** a Structural Heart Program, St Michael's Hospital, University of Toronto, Toronto, Ontario, Canada; b Electrophysiology Department, University Health Network, University of Toronto, Toronto, Ontario, Canada

**Keywords:** pacemaker leads, transcatheter tricuspid valve replacement, tricuspid regurgitation

## Abstract

**Background:**

Cardiac implantable electronic device (CIED)–related tricuspid regurgitation (TR) is increasingly recognized, necessitating heart team assessment. Transcatheter tricuspid valve replacement (TTVR) is emerging as an option for inoperable patients with TR, requiring decisions on right ventricular lead management.

**Case Summary:**

A 76-year-old woman developed moderate to severe TR after pacemaker implantation, which was exacerbated by a second RV lead 8 years later, leading to torrential TR and recurrent heart failure hospitalizations. TR persisted despite lead extraction, and multimodality imaging revealed complex valve anatomy. TTVR was performed, yielding excellent echocardiographic results and improved quality of life.

**Discussion:**

CIED and TR can coexist, obscuring the distinction between lead-related and lead-associated TR because of right ventricular remodeling. The heart team should determine lead management during transcatheter procedures. TTVR is a valuable option for massive gaps, severe tethering, or complex anatomy.

**Take-Home Message:**

Collaborative management with electrophysiology as part of the extended heart team is key in CIED-related TR cases.

## Graphical Abstract

**Figure:**
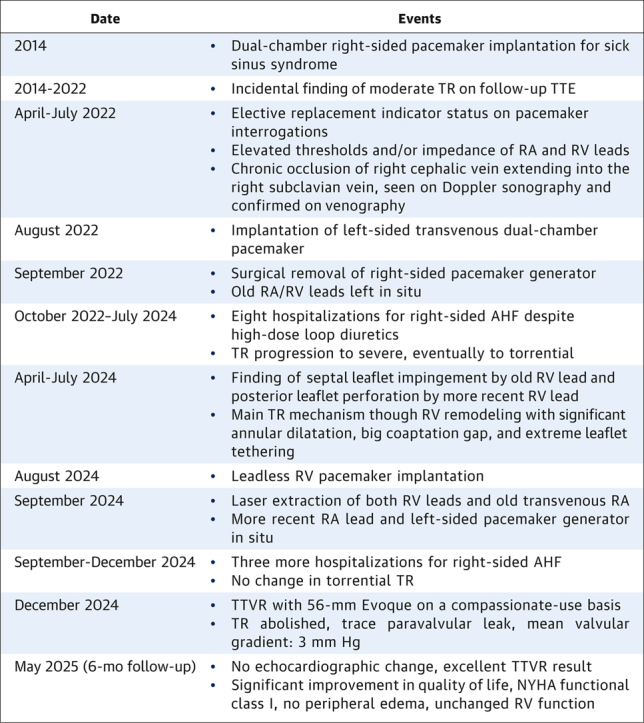

## History of Presentation

A 76-year-old woman with recurrent hospitalizations for acute heart failure and severe tricuspid regurgitation (TR) was referred to our structural clinic for transcatheter tricuspid valve intervention (TTVI) assessment. She presented with massive bilateral peripheral edema, a 4/6 holosystolic murmur at the left lower sternal border, and NYHA functional class III dyspnea.

## Past Medical History

**Figure 1:**
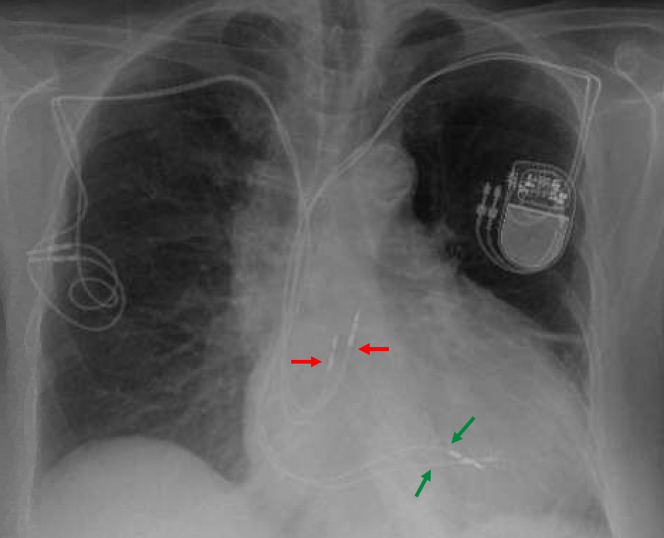
Chest X-Ray After Contralateral Generator Replacement and Implantation of 2 New Leads Anteroposterior chest x-ray after left-sided dual chamber pacemaker replacement and right-sided generator removal showing a total of 2 right atrial (red arrows) and 2 right ventricular (green arrows) leads.

The patient had a history of congestive heart failure, permanent atrial fibrillation, hypertension, and ulcerative colitis. Ten years prior, owing to sick sinus syndrome and syncopal episodes, she underwent dual-chamber pacemaker implantation via the right cephalic vein. During the next 8 years, up to the point of elective pacemaker generator replacement, she had no hospitalizations for her acute heart failure and was found to have moderate TR and right ventricular (RV) dilatation, although further etiological evaluation was not undertaken. As both the right atrial and RV leads had elevated pacing thresholds and/or impedance, and the right-sided venous system was occluded, a new dual-chamber pacemaker was implanted via the left cephalic vein. Two weeks later, the right-sided generator was removed, although the old leads were left in place (Figure 1). Shortly after, she was hospitalized for right-sided acute heart failure. Over a span of 2 years, 7 more hospitalizations at tertiary-care hospitals followed, despite high-dose diuretics (160 mg furosemide daily). Also, progression of TR from moderate to severe was noticed, which finally warranted referral to our structural clinic.

## Differential Diagnosis

**Figure 2:**
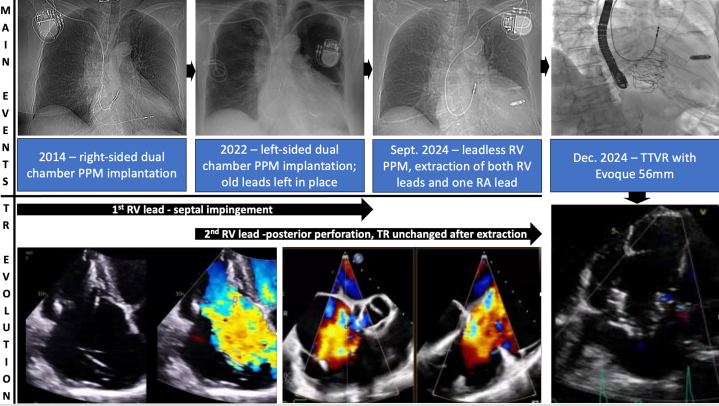
From CIED-Related Tricuspid Regurgitation to TTVR

The differential diagnosis included functional atrial TR due to permanent atrial fibrillation, functional ventricular TR due to right-heart remodeling, and also cardiac implantable electronic device (CIED)–related TR (Figure 2).

## Investigations

**Figure 3:**
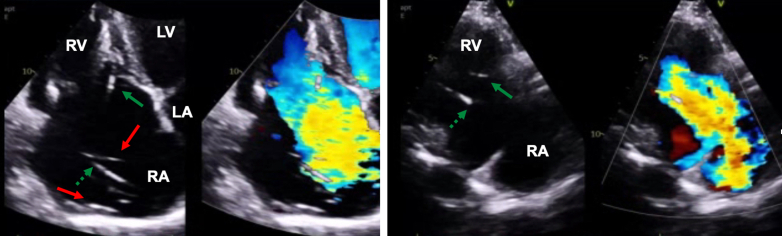
Transthoracic Echocardiographic Assessment of Tricuspid Regurgitation Apical 4-chamber view (left panel) and right-sided 2-chamber view (right panel) showing torrential tricuspid regurgitation with main central jet and annular and right ventricular dilatation. Notice the presence of 2 right atrial leads (red arrows) and 2 right ventricular leads with different course, one anteroseptal (solid green arrow) versus one posterior (dashed green arrow); see also Video 1. LA = left atrium; LV = left ventricle; RA = right atrium; RV = right ventricle.

**Figure 4:**
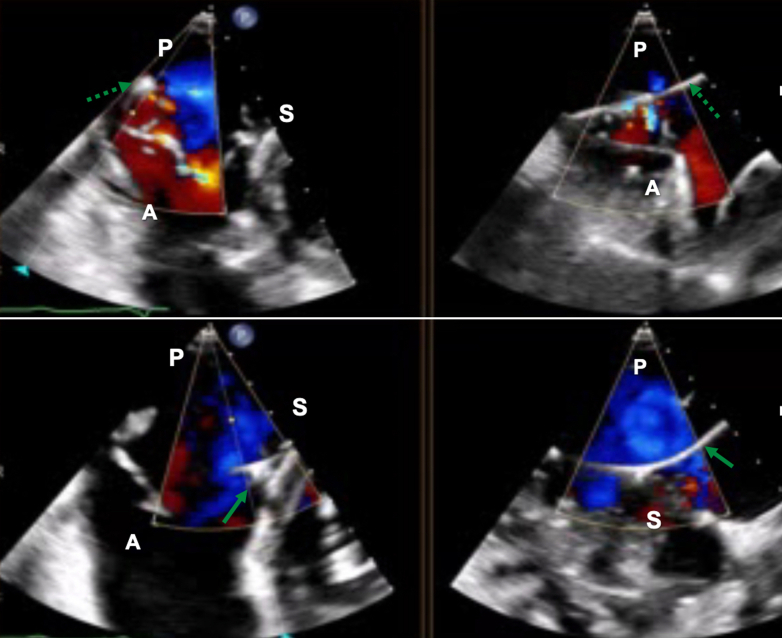
Transesophageal Echocardiographic Assessment of Tricuspid Regurgitation Transesophageal echocardiography showing biplane transgastric en face images of the tricuspid valve, focusing on both the anteroposterior (upper panel) and anteroseptal commissure (lower panel), with evidence of septal leaflet impingement by one of the 2 right ventricular leads (solid green arrow) and posterior leaflet perforation by the other lead (dashed green arrow). A = anterior; P = posterior; S = septal.

Blood work revealed N-terminal pro–B-type natriuretic peptide 4,651 pg/mL, glomerular filtration rate 50 mL/min, and bilirubin 3.63 mg/dL; electrocardiogram showed atrial fibrillation with paced ventricular rhythm. Right and left heart catheterization ruled out coronary artery disease and pulmonary hypertension (mean pulmonary arterial pressure: 18 mm Hg) but showed ventricularized right atrial pressure form (mean pressure: 14 mm Hg, V-wave: 20 mm Hg). Transthoracic echocardiography (TTE) revealed torrential TR due to annular tricuspid valve (TV) dilatation with huge coaptation gap, but also possible pacemaker lead impingement (Figure 3, Video 1). Transesophageal echocardiography (TEE) confirmed impingement of TV septal leaflet by 1 of the 2 RV leads. However, the second RV lead was restricting motion of the TV posterior leaflet by perforation (Figure 4, Video 2), so both were contributing to the TR mechanism, on top of the underlying functional etiology. Of note, left ventricular ejection fraction was normal, and there was no significant left-sided valvular disease.

## Management

**Figure 5:**
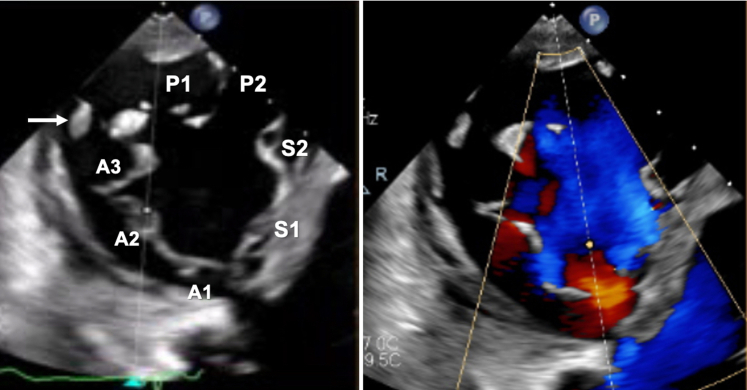
Tricuspid Valve Morphology Transgastric en face views of the tricuspid valve showing a 7-scallop valve, with severe leaflet tethering (left panel) causing torrential tricuspid regurgitation (right panel). The white arrow indicates the anteroposterior papillary muscle. A = anterior; P = posterior; S = septal.

**Figure 6:**
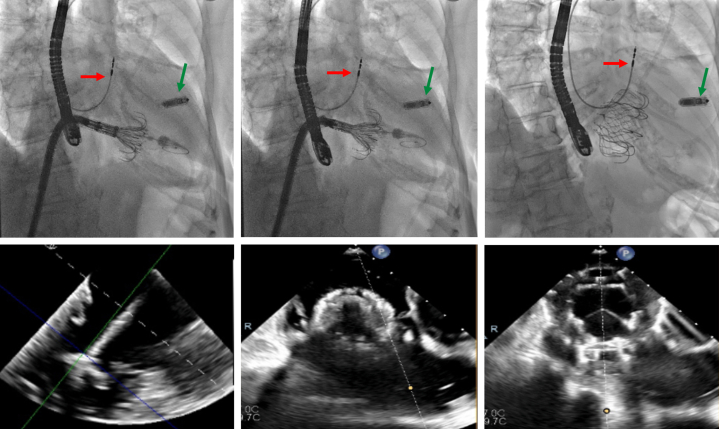
Transcatheter Tricuspid Valve Replacement Steps Fluoroscopic (upper panel) and equivalent echocardiographic (lower panel) steps for orthotopic transcatheter tricuspid valve replacement with the Evoque system. The red arrow indicates the remaining right atrial lead, the green arrow indicates the leadless right ventricular pacemaker.

The case was discussed by the heart team, and a decision to address the CIED-related TR component was made. Therefore, a leadless RV pacemaker (Micra, Medtronic) was first implanted, then both RV leads and the right-sided RA lead were extracted with laser. Unfortunately, no change in TR severity or symptoms was noticed, and 2 more acute heart failure hospitalizations followed over the next 3 months. Repeat TTE and TEE also showed RV dilatation (basal diameter: 55 mm) and systolic impairment (tricuspid annular plane systolic excursion: 13 mm) (Video 3), and the unique TV morphology with 7 leaflet scallops (3× anterior, 2× septal, 2× posterior) could finally be appreciated without the RV leads in place (Figure 5, Video 4). Considering the patient's STS score of 5.3%, TriSCORE^1^ of 7 (34% predicted in-hospital postsurgical mortality), age, and frailty, the heart team considered her inoperable and recommended TTVI. Owing to extreme leaflet tethering and coaptation gap of 22 mm, transcatheter edge-to-edge repair was not an option,^2^^,^^3^ so the patient underwent computed tomography screening for orthotopic TTVR. With a TV annulus of 51.2 × 47 mm at end-diastole and a perimeter-derived diameter of 50.6 mm, the patient qualified for a 56-mm Evoque device (Edwards Lifesciences)^4^ on a compassionate-use basis. After preclosing the right femoral vein with 2 Prostyle devices (Abbott), we used an Agilis steerable catheter (Abbott) to place a Safari extra-small wire (Boston Scientific) in the RV apex, confirming a chord-free path. Under echocardiographic and fluoroscopic guidance, we implanted the 56-mm Evoque valve (Figure 6, Video 5), with stable position, no residual TR, unchanged moderate RV dysfunction, and unaltered ventricular pacing.

## Outcome and Follow-Up

After TTVI, the patient was discharged in stable condition on postoperative day 3. At the 6-month follow-up, TTE showed a well-seated bioprosthetic Evoque valve, with mean gradient of 3 mm Hg at a heart rate of 58 beats/min, trace intravalvular prosthetic regurgitation, trace paravalvular leak (Video 6), and still moderate systolic RV impairment. Most importantly, the patient reported significant improvement to NYHA functional class I, with stable weight and a 75% reduction in her daily furosemide requirement (now 40 mg daily).

## Discussion

CIED-related TR is increasingly recognized as an independent clinical entity,^5^ usually arising from mechanical interference with the TV apparatus.^6^^,^^7^ As transcatheter TR treatment options continuously evolve, the correct management of leads impairing the TV closing mechanism needs to be discussed by the extended heart team, including electrophysiologists, on a case-by-case basis. Although transvenous lead extraction may seem compelling, it can pose risks, including damage to the TV apparatus^8^ or even further TR worsening.^9^ Moreover, CIED and functional TR may coexist in many patients, so TR improvement may not occur after lead extraction, particularly in the presence of significant RV and TV annular remodeling.^10^ On the other hand, jailing a lead into a commissure or between 2 clips during edge-to-edge repair, or jailing it with an orthotopic valve during TTVR, might cause lead dysfunction. This could pose significant problems for patients dependent on ventricular pacing.

This case report highlights the need for comprehensive understanding of the TR mechanism, especially in the presence of CIED. When causality between TR appearance or worsening and recent RV lead insertion can be established, CIED-related TR needs to be addressed promptly by lead extraction.^11^ However, CIED and TR may coexist for years and obscure the differentiation between lead-related and lead-associated^5^ TR because of the predominance of RV and TV annular remodeling. This was the case for our patient, as the first RV lead had an anteroseptal path and impinged on the septal leaflet, causing moderate TR. Eight years later, the second RV lead put the final nail in the coffin by posterior leaflet perforation, with further TR and RV worsening. In hindsight, a more thorough search for the root cause of TR should have been conducted after the first permanent pacemaker implantation. Abandoning the leads after generator replacement certainly did not help, but annular dilatation and right heart remodeling had already taken place by that time.

With the emergence of TTVR as a valuable therapeutic option in symptomatic inoperable TR patients^4^ unsuitable for other TTVI measures, whether to extract or jail RV leads needs to be decided by interventional cardiologists, electrophysiologists, and cardiac surgeons on an individual basis. Lead jailing is usually preferred in multimorbid elderly patients, particularly if they are not pacer dependent and if severe annular dilatation has already occurred. On the other hand, transvenous RV lead extraction has an acceptable risk-benefit ratio, occasionally improves TR severity, and may facilitate TR intervention in selected cases. Such patients will require pacing alternatives such as leadless pacemakers or coronary sinus leads.

## Conclusions

Orthotopic TTVR is emerging as the go-to option in inoperable TR patients with massive coaptation gaps, severe leaflet tethering, or complex anatomy, even in the presence of CIED-related or CIED-associated TR.

**Take-Home Messages**

• Lead-related TR is progressive, may go undiagnosed for years, and may be difficult to distinguish from lead-associated TR because of chronic right-heart remodeling.
• Collaborative management with electrophysiology as part of the extended heart team is key to optimal clinical outcomes.
• In elderly comorbid inoperable patients, TTVR is feasible either by jailing leads or after transvenous lead extraction.
• Massive coaptation gaps and tricuspid valve leaflet tethering are best treated with TTVR.

**Visual Summary:** Timeline of Key Events

Date	Events
2014	• Dual-chamber right-sided pacemaker implantation for sick sinus syndrome
2014-2022	• Incidental finding of moderate TR on follow-up TTE
April-July 2022	• Elective replacement indicator status on pacemaker interrogations • Elevated thresholds and/or impedance of RA and RV leads • Chronic occlusion of right cephalic vein extending into the right subclavian vein, seen on Doppler sonography and confirmed on venography
August 2022	• Implantation of left-sided transvenous dual-chamber pacemaker
September 2022	• Surgical removal of right-sided pacemaker generator • Old RA/RV leads left in situ
October 2022–July 2024	• Eight hospitalizations for right-sided AHF despite high-dose loop diuretics • TR progression to severe, eventually to torrential
April-July 2024	• Finding of septal leaflet impingement by old RV lead and posterior leaflet perforation by more recent RV lead • Main TR mechanism though RV remodeling with significant annular dilatation, big coaptation gap, and extreme leaflet tethering
August 2024	• Leadless RV pacemaker implantation
September 2024	• Laser extraction of both RV leads and old transvenous RA • More recent RA lead and left-sided pacemaker generator in situ
September-December 2024	• Three more hospitalizations for right-sided AHF • No change in torrential TR
December 2024	• TTVR with 56-mm Evoque on a compassionate-use basis • TR abolished, trace paravalvular leak, mean valvular gradient: 3 mm Hg
May 2025 (6-mo follow-up)	• No echocardiographic change, excellent TTVR result • Significant improvement in quality of life, NYHA functional class I, no peripheral edema, unchanged RV function

AHF = acute heart failure; RA = right atrium; RV = right ventricle; TR = tricuspid regurgitation; TTE = transthoracic echocardiography; TTVR = transcatheter tricuspid valve replacement.

## Funding Support and Author Disclosures

Funded by the Deutsche Forschungsgemeinschaft (DFG, German Research Foundation), project number 545263014. Dr Fam has been a consultant to Edwards Lifesciences, Abbott, Cardiovalve, Medtronic, Tricares, inQB8, and Jenscare. All other authors have reported that they have no relationships relevant to the contents of this paper to disclose.

## Appendix

Video 1 Transthoracic Echocardiographic Assessment of Tricuspid Regurgitation Apical 4-chamber and modified right-sided 2-chamber view showing torrential tricuspid regurgitation with main central jet and annular and right ventricular dilatation. Notice the presence of 2 right atrial leads and 2 right ventricular leads with different course, one anteroseptal versus one posterior.

Video 2 Transesophageal Echocardiographic Assessment of Tricuspid Regurgitation Transesophageal echocardiography showing biplane transgastric en face views of the tricuspid valve, shifting from the anteroposterior to the anteroseptal commissure, with evidence of septal leaflet impingement by one of the 2 right ventricular leads and posterior leaflet perforation by the other lead.

Video 3 Transthoracic Echocardiographic Assessment of Tricuspid Regurgitation After Extraction of Both Right Ventricular Leads Parasternal and apical right-sided 2-chamber views showing unchanged torrential tricuspid regurgitation due to severe right ventricular remodeling and tricuspid valve annular dilatation.

Video 4 Transesophageal Echocardiographic Assessment of Tricuspid Regurgitation After Extraction of Both Right Ventricular Leads Transgastric and midesophageal en face and inflow views showing unchanged torrential tricuspid regurgitation due to severe leaflet tethering, right ventricular remodeling, and tricuspid valve annular dilatation. Notice the abundance of tricuspid valve scallops: 3 anterior, 2 posterior, and 2 septal in total.

Video 5 Transcatheter Tricuspid Valve Replacement Steps Transesophageal multiplanar reconstruction of midesophageal views showing the procedural steps and final fluoroscopic result of orthotopic transcatheter tricuspid valve replacement with the Evoque system.

Video 6 Results of 6-Month Echocardiographic Follow-Up Transthoracic parasternal and apical views showing well-seated bioprosthetic Evoque valve with trace intravalvular prosthetic regurgitation and trace paravalvular leak.
